# Mechanical Characterization of FDM 3D-Printed Components Using Advanced Measurement and Modeling Techniques

**DOI:** 10.3390/ma18051086

**Published:** 2025-02-28

**Authors:** Marcin Wikło, Bartłomiej Henryk Byczuk, Kinga Skrzek

**Affiliations:** 1 The Faculty of Mechanical Engineering, Casimir Pulaski University of Radom, Stasieckiego 54 St., 26-612 Radom, Poland; 2 Vesuvius Sp. z o.o., Jasnogórska 11, 31-358 Krakow, Poland; bartlomiej.byczuk@vesuvius.com; 3 The Faculty of Mechanical Engineering, Wrocław University of Science and Technology, Łukasiewicza 5 St., 50-370 Wroclaw, Poland; kinga.skrzek@pwr.edu.pl

**Keywords:** 3D printing, tensile testing, FDM parameters, material characterization, DIC, FEMU

## Abstract

The study investigates the mechanical characterization of PET-G components fabricated via Fused Deposition Modeling (FDM), integrating experimental testing with advanced numerical modeling. Initially, an extensive parametric analysis was conducted to determine the optimal printing conditions, focusing on temperature, speed, and infill density to ensure reliable and repeatable sample fabrication. Subsequently, the study employs an inverse problem-solving approach that combines Digital Image Correlation (DIC) with Finite Element Method Updating (FEMU) to identify the material parameters, specifically Young’s modulus and Poisson’s ratio. The methodology allows for a precise evaluation of mechanical properties by iteratively minimizing discrepancies between experimental strain fields and FEM simulations. The results reveal significant dependencies of material stiffness on infill pattern and density, with Young’s modulus varying up to 20% between different configurations. Additionally, the study highlights the limitations of conventional tensile testing for FDM materials, emphasizing the necessity for advanced full-field measurement techniques to account for anisotropy and microstructural heterogeneity. The proposed methodology enhances the accuracy of material characterization, contributing to the development of more reliable predictive models for 3D-printed components. The research provides valuable insights for optimizing FDM process parameters and establishing standardized testing protocols for additively manufactured materials.

## 1. Introduction

Additive manufacturing is one of the pillars of Industry 4.0. A growing number of new production fields in which 3D printing is used, along with the expansion of technologies and 3D printing materials, including printing from concrete [1,2,3], high-strength laminates [4] or even electronics with sensors [5,6,7] shows that additive production is a dynamically developing and very promising part of the different kinds of industries. One of the advantages of 3D printing is the ability to produce ready-to-use elements with complex geometries, which can be used to reduce the weight of elements, manufacturing cost and re-use of plastic waste, which positively affects a sustainable economy [8,9].

One of the most commonly used 3D printing technologies is Fused Deposition Modelling (FDM), which relies on the layer-by-layer deposition of thermoplastic material in the form of filaments. While FDM offers numerous advantages, one of the key challenges remains the accurate determination of the mechanical strength of printed parts, which is crucial for many applications where reliability and durability are essential. The FDM process is characterized by anisotropy in mechanical properties, meaning that the strength of a part can vary significantly depending on the printing direction and process parameters. Therefore, it is important to understand how different factors, such as printing orientation, extruder temperature, print speed, layer thickness, and infill density, influence the final mechanical properties of printed parts [10,11]. Research on the mechanical properties of FDM prints focuses on analysing the impact of process parameters on strength, stiffness, and other mechanical characteristics of materials. For example, studies on the influence of print orientation and process parameters on the thermal and mechanical behaviour of ULTEM^®^ 9085 have shown that these parameters can significantly affect the final strength of parts [12]. Research on optimal printing conditions for producing strong polycarbonate components has shown that variations in process parameters can lead to significant differences in strength [13,14,15].

Mechanical anisotropy, resulting from the characteristic layer-by-layer deposition process in FDM technology, has been extensively studied in the context of tensile and compressive strength, which can differ by up to 50% depending on the printing orientation [14,16]. Similarly, the influence of additives on the anisotropic strength of ABS has been demonstrated, showing that material modifications can partially reduce these differences [17].

One of the critical aspects affecting the strength of FDM prints is internal porosity and the quality of layer bonding. Studies indicate that process parameters such as temperature and print speed can influence the degree of infill and porosity, which in turn affects the final mechanical strength of parts [18,19,20].

The colour of the material and its modifications can also impact the mechanical properties of prints. For instance, studies on the influence of PLA colour on mechanical properties have shown that differences in pigments can affect the tensile strength and flexibility of parts [21]. Additionally, research on hybrid polymer-metal materials suggests the potential for improving mechanical properties through appropriate material combinations [22]. A comprehensive review of the impact of FDM process parameters on the mechanical strength of polymers highlights the need for further research on optimizing these parameters [23].

Issues related to the selection of appropriate materials for FDM printing are also crucial from the perspective of mechanical strength. A literature review on challenges associated with materials used in 3D printing technology emphasizes the need for continuous improvement. Studies on the mechanical properties of FDM parts described by classical laminate theory highlight the importance of optimizing layer orientation [24]. Experimental characterization and modelling of the mechanical properties of parts made from ABS indicate that the optimization process can significantly improve the strength of elements [25]. The measurement of anisotropic compressive strength in prototype parts confirms the importance of understanding this phenomenon in the design of 3D prints [26].

Research on the parametric influence of the FDM process on the mechanical strength of parts suggests that optimizing print parameters can greatly enhance outcomes [27]. In a similar vein, studies on the performance of interlayer bonds in continuous fibre-reinforced composites printed by FDM indicate potential opportunities for part reinforcement [28] and with connection of the optimal topology design [29,30,31].

Studies on the impact of infill design on the cost and production time of ABS prints show that appropriate infill configuration can influence production efficiency and mechanical properties [32]. Modelling the bonding formation between polymer filaments in the FDM process is crucial for understanding the mechanisms responsible for part strength [11,33].

The anisotropic mechanical properties of parts made using FDM are a significant contribution to understanding the complex properties of these materials [16,34]. An analysis of the impact of various raster angles on the mechanical properties of ABS prints indicates the possibility of improving strength through appropriate optimization of these parameters [27,35].

Recent advancements in Artificial Intelligence (AI) have introduced innovative methodologies in the field of 3D printing. AI-driven techniques are increasingly being utilized to optimize material selection for specific applications, such as medical devices [36] Furthermore, AI facilitates the systematic investigation of complex interactions between intrinsic material properties and processing parameters in biocomposites [37], enabling enhanced predictive modelling and process control.

The precise identification of mechanical parameters in 3D-printed components presents a notable challenge, especially when considering complex and heterogeneous material systems. Conventional material characterization techniques predominantly utilize universal testing machines, outfitted with load cells and electrical extensometers or strain gauges. These approaches necessitate an a priori understanding of stress distribution within the specimen and are typically classified as “statistically determinate” methodologies. Conversely, contemporary advancements have emphasized the utility of inverse methods for material property characterization. These methods, which capitalize on full-field experimental data, enable the identification of material parameters through the optimization of model predictions against observed behaviors. Such approaches are particularly efficacious when applied to complex material models, including composites [38,39,40], brittle materials [41], and components fabricated via additive manufacturing. Nonetheless, the predominant focus of research for 3D printing components has been the structural behavior of these parts [42] rather than the direct determination of their constitutive model or failure behavior [43].

Within the realm of inverse methods, two approaches have emerged as preeminent: the Virtual Fields Method (VFM) [44] and Finite Element Method Updating (FEMU) [45]. These methodologies are widely regarded for their robustness in facilitating the identification of material models. Despite their potential, full-field techniques are not without limitations, including significant computational demands and susceptibility to experimental noise [46,47]. Despite these drawbacks, research [47] advocates that camera-based full-field measurement techniques represent a paradigm shift in materials science research. By enabling more detailed and accurate analyses of material properties, these advancements hold great promise for developing next-generation materials with tailored properties.

Despite the extensive research on the mechanical properties of FDM-printed components, most studies rely on conventional tensile testing methods that assume homogeneity and uniform stress distribution. The study introduces a novel approach by integrating Digital Image Correlation (DIC) with an inverse problem-solving methodology based on Finite Element Method Updating (FEMU). This combined technique enables precise identification of material parameters, including Young’s modulus and Poisson’s ratio, by minimizing discrepancies between full-field experimental strain measurements and numerical simulations. Unlike standard mechanical testing, which is limited to predefined cross-sectional assumptions, the proposed method captures the compound nature of 3D-printed PET-G structures, providing a more accurate and comprehensive material characterization.

## 2. Testing and Results

The preliminary stage of this investigation was dedicated to identifying the optimal additive manufacturing parameters for the materials under study, with particular emphasis on the interplay between printing temperature and speed. The primary focus of the subsequent research phase, as elaborated in the following chapter, was a comprehensive evaluation of the mechanical response of these materials under tensile loading conditions. This assessment incorporated the use of probes with modified thicknesses, analysed through the application of an advanced inverse problem framework, which synergistically integrates Digital Image Correlation (DIC) techniques with Finite Element Method (FEM) simulations to ensure robust and precise mechanical material identification.

The research was centred around a particular material PET-G (glycol-modified polyethene terephthalate), which is quite easy to print. PET-G offers high mechanical strength, as stated by filament manufacturers, and allows the printed part to operate at elevated temperatures. The properties of the selected filament are listed in Table 1.

All studies were conducted using material sourced from the same batch from a single manufacturer to avoid variations in the mechanical properties. Transparent PET-G filament was chosen to eliminate variables related to additives or dyes present in other blends. To ensure repeatable tests, the filament was stored in a dedicated desiccator/heater unit to keep humidity absorption low and to perform a beneficial preheat. A pneumatic hose connected the chamber to a filament dryer, ensuring precise testing conditions.

The samples were made using FDM technology with the Kingroon KP3S 3D printer. The printer has a working area of 180 × 180 × 180 mm. The mounted hotend has a brass nozzle with orifice diameter of 0.4 mm. The 3D printer has a heated work table that allows it to warm up to 110 °C. The maximum 3D printing speed is 200 mm/s.

### 2.1. Determination of the Optimal Printing Parameters

#### 2.1.1. Temperatures

Each material used in 3D printing has certain plasticization temperature limits; the selected PET-G values are shown in Table 1. To determine the effect of extrusion temperature on the actual print volume, an extended temperature range starting from 210 °C with steps of 5 degrees up to 240 degrees was adopted as shown in Table 2. Extrusion length (theoretical length of extruded material) was equal to L=100 mm, extrusion speed $V_{W}=150\;\frac{\mathrm{mm}}{\min}$, and diameter of extruder nozzle to 0.4 mm. The information on whether the material was extruded in its entirety and whether the extruder knurl did not damage the surface of the extruded material is also verified.

**Figure 1:**
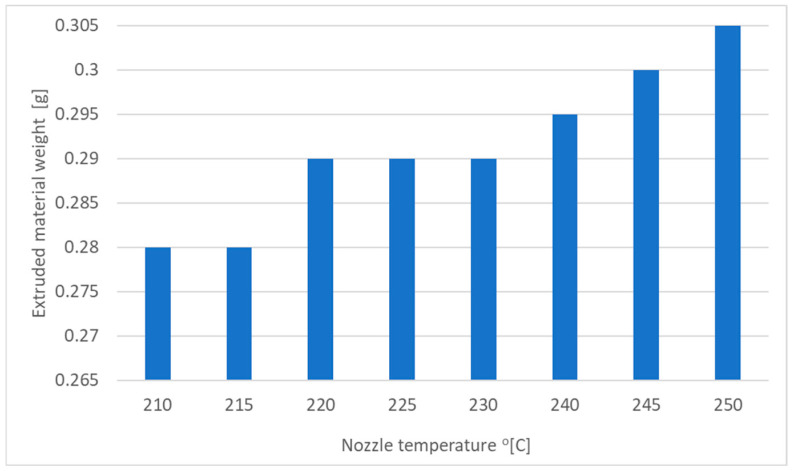
Dependence of the extruded material weight in the function of the temperature.

As can be seen in the Figure 1, the increase in nozzle temperature affects the amount of material extruded. This is due to the decrease in material viscosity with increasing temperature. It should be noted that temperatures close to maximum can cause additional stress and degradation of the printed material.

#### 2.1.2. Printing Speed

The design of the extrusion head enables adjustment of the extruder knurl speed. The trial pertains to modifying the printing speed while maintaining a consistent temperature. Although the chosen printer model does not prescribe specific maximum print speed values, empirical evidence and extruder tests indicate a prudent speed range from 150–500 mm/min. The trial was conducted within this specified range, with speed increments set at 50 mm/min and the results were gathered in Table 3.

**Figure 2:**
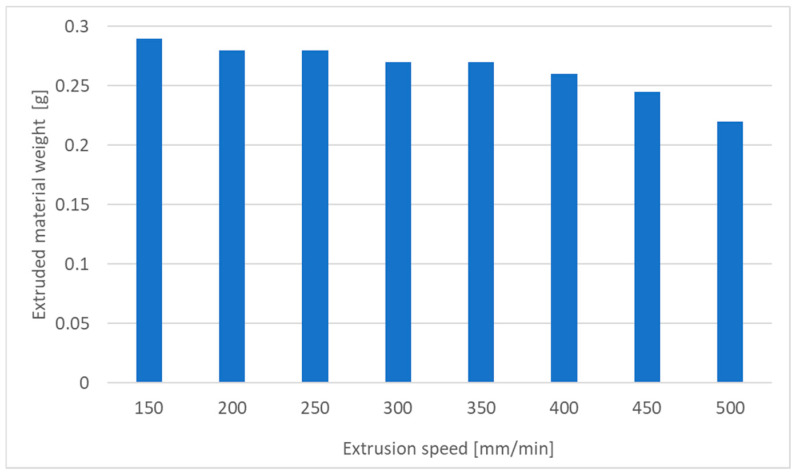
Dependence of the extruded material weight in the function of the extrusion speed.

Figure 2 shows that at the speed of 250 mm/min, the amount of material extruded significantly decreases. This is related to the increase in resistance and the possibility of plasticizing the material by the head. For speeds of 450 and 500, the skipping of the knurls was audible. This determines the maximum printing speed for a given temperature.

#### 2.1.3. Mechanical Properties of the Various Kinds of Pattern

One of the most challenging questions during the preparation of the mechanical component print is what printing parameters should be considered and what kind of internal structure should be used to achieve the highest strength. To find the answers to the stated problem and define the mechanical properties of the material, a series of static tensile tests were performed. The probes were prepared with different infill types most often available to users, which are listed in Table 4.

The division of groups is as follows: samples 1–14 are grouped according to variable fill type; samples 15–23 (including 6) are grouped according to sample fill level; samples 24–27 (including 6) are grouped according to print layer height; samples 28–33 (including 6) are grouped according to print temperature; and samples 34–35 and 6 are grouped according to print speed.

Samples were made according to the standard recommendations, Plastics—Determination of tensile properties ISO 527 [49], with the dimensions: $L_{3}=150\;[mm]$—overall length, $L_{2}=106\;[mm]$—distance between broad parallel-sided portions, L=115 [mm]—initial distance between grips, $L_{0}=50\;[mm]$—gauge length, R=78 [mm], $B_{2}=20\;[mm]$—width at ends, $B_{1}=10\;[mm]$—width of narrow portion, h=4 [mm]—thickness.

Solid models of the tested samples were made with Inventor Professional. In order to obtain accurate test results, six samples per test as specified in Table 4 were printed in a single setup. Table 5 presents the optimal determined parameters of the 3D printing process based on the previous tests. The model was prepared using the slicer program Ultimaker Cura.

**Figure 3:**
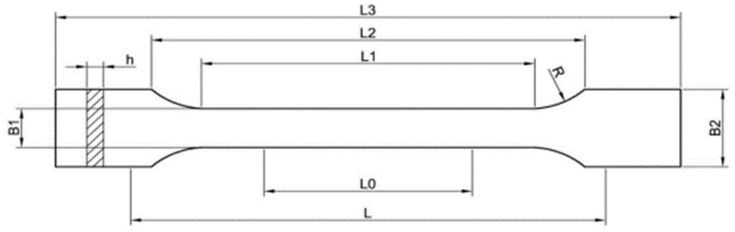
View of test specimen [49].

The printed samples underwent static tensile testing, a fundamental method for determining a material’s mechanical properties. This test involves the axial stretching of samples with precisely defined dimensions and shapes, securely held in the grips of the testing machine. The dimensions were selected according to the ISO 527-1 standard, as shown in Figure 3, which outlines acceptable sample specifications for plastics. The testing process continues until the specimens fracture, with both force and displacement recorded.

**Figure 4:**
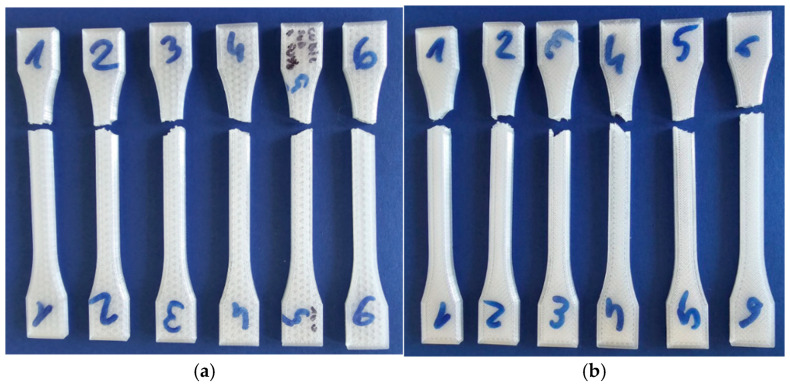
Samples after uniaxial stretching for probes: (**a**) cubic subdivision (see Table 4 pos.5) and (**b**) ZigZag (see Table 4 pos.14).

**Figure 5:**
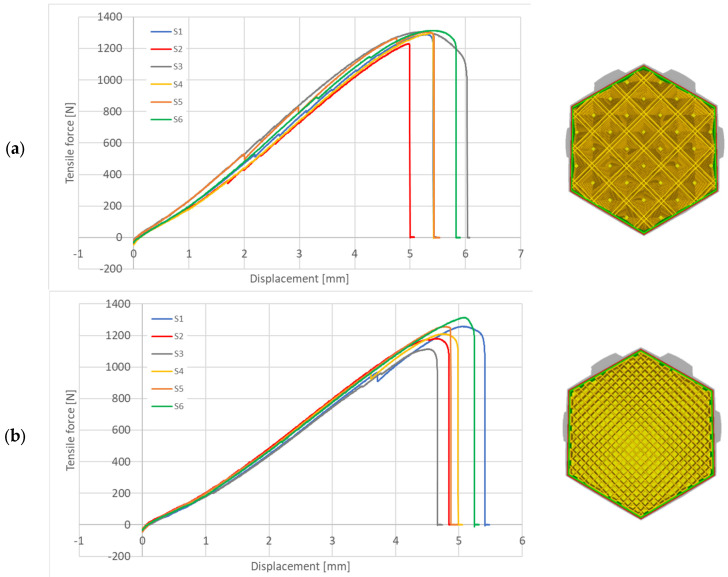
Tensile force-displacement relationship for different infill patterns. (**a**) Cubic Subdivision pattern (see Table 4 pos.5) and (**b**) ZigZag pattern (see Table 4 pos.14).

**Figure 6:**
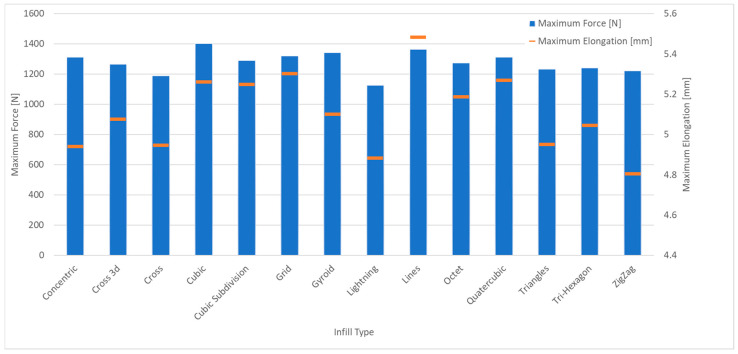
Maximum breaking force and elongation for various kinds of infill.

Post-uniaxial stretching samples are depicted in Figure 4, revealing a similar fracture pattern and force curve shape as illustrated in Figure 5. The article presents two exemplary series: the cubic subdivision in Figure 5a and the ZigZag pattern in Figure 5b. The resulting curves illustrate the relationship between displacement and force, while Figure 6 compares the maximum breaking forces across all research series.

The stretching plots presented in Figure 5 exhibit distinct characteristics attributed to the variability in specimen printing and the non-uniform properties of the filament used. These factors have led to observable discrepancies in the mechanical performance of some samples, including instances of rapid failure. Notably, the maximum breaking force varied significantly, with differences reaching up to 15% across the tested specimens.

The biggest difference in all sample series is the elongation of the samples. Worth noting is the difference in maximum force used to break the samples, all of the infills span around 250 N. This can indicate that the type of infill mostly focuses itself on stretch of the part, as the infill pattern fibres are placed differently, and this strongly changes maximum adhesion forces in the printed sample.

**Figure 7:**
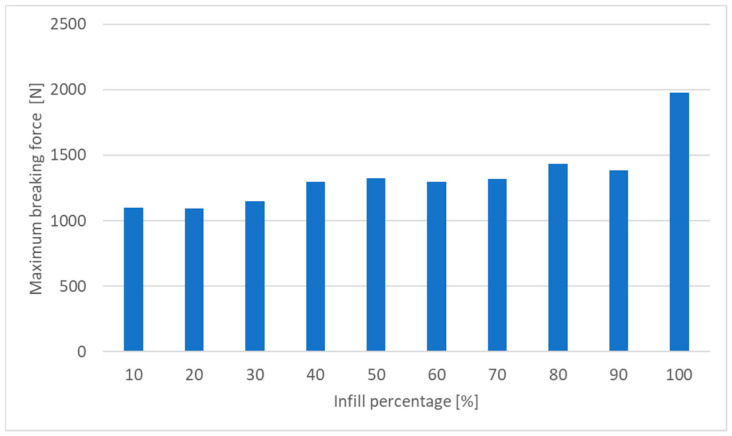
Changes in the breaking force in the function of the infill percentage.

The maximum breaking force shown in Figure 7 highly depends on the infill percentage. The difference in the force between 10% and 100% of infill is almost 800 N. In comparison, a slight change in the breaking force is noted in the range of 40% to 90%.

**Figure 8:**
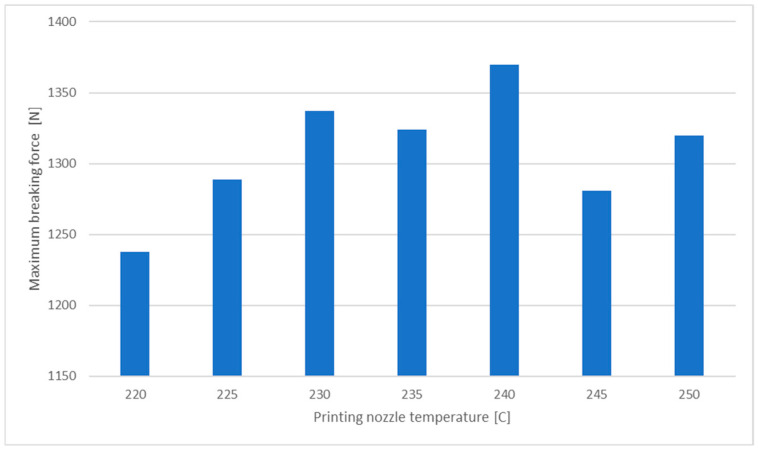
Changes in the breaking force in the function of the printing temperature.

When analysing the relationship between breaking force and printing nozzle temperature (Figure 8), it becomes evident that maintaining the midpoint of the recommended printing temperature range (see Table 1) represents the most favourable choice. It is within this range that the highest force values are consistently observed.

#### 2.1.4. Modification of the Sample Thickness

The probe’s thickness depicted in Figure 3 plays a crucial role in influencing the model parameters, with particular relevance in studies adhering to standardized protocols that define the thickness as 4 mm. This parameter is especially critical in additive printed component testing, where the infill structure is enclosed by external walls, and its influence on the overall material behaviour remains substantial. Consequently, determining the optimal probe thickness is a prerequisite for subsequent research stages.

The methodological framework for this investigation entails systematically varying the probe thickness within a range of 4 mm to 10 mm. This approach includes evaluating three distinct infill patterns, selected based on their significant differences in performance in the preceding experimental phase, to ensure representative values for all infills.

**Figure 9:**
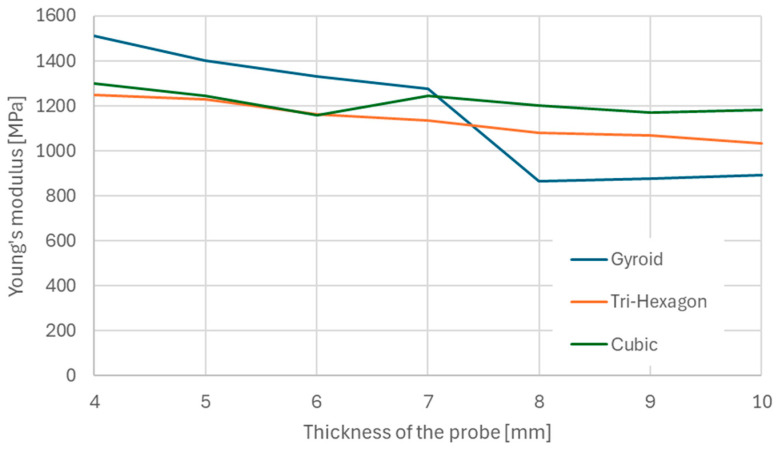
Change of the Young’s modulus due to increase of the probe thickness.

The variation in the material’s elasticity modulus, as shown in Figure 9 decreases as a function of probe thickness, stabilizing at 8 mm. The thickness modification results in a significant reduction of the modulus from 1510 MPa to 890 MPa. This 58% reduction underscores the importance of considering probe thickness carefully prior to final testing.

### 2.2. Determination of the Mechanical Parameters Defined as the Inverse Problem

#### Definition of the Inverse Problem

One of the most significant challenges in model parameter identification lies in defining material parameters that are highly dependent on printing parameters, infill density, and pattern. Traditional testing procedures utilize force data from the testing machine along with deformation measurements either from the machine or an extensometer. This approach requires prior knowledge of the cross-sectional area of the probe, which for printed materials is often unknown. Additionally, it limits the analysis to a single measured strain. To address these limitations, this research proposes a methodology for material parameter identification, the Young Modulus along with the Poisson ratio, based on an inverse problem approach, integrating Finite Element Method (FEM) simulations with Digital Image Correlation (DIC) techniques.

The experimental procedures were executed utilizing the ZWICK/ROEL universal static testing machine to ensure high-precision mechanical characterization. Tensile testing was conducted in strict accordance with ISO 527 standards, maintaining all recommended testing parameters to uphold methodological rigor. Strain field measurements were obtained through the ZEISS DIC adjustable modular camera system ARAMIS, comprising two high-resolution 24-megapixel cameras, with data acquisition at a temporal resolution of 5 Hz. The deformation analysis was carried out using the ZEISS INSPECT Correlate 2023 SP3, which facilitated comprehensive strain evaluation. To enhance measurement accuracy, the DIC system was synchronized with the force signal from the testing machine, enabling real-time correlation of strain evolution with the applied tensile load.

The problem was formulated as a multi-objective optimization task, with objective functions defined as follows:

(1) $$f_{i}^{c}(E,\upsilon )={\left(\epsilon_{i}^{FEM}(E,\upsilon )-\epsilon_{i}^{exp}\right)}^{2}$$

where the $\epsilon_{i}^{FEM}$ represents the strain from the FEM simulation and $\epsilon_{i}^{exp}$ denotes the strain from experimental measurements. The superscript i corresponds to the number of the objective function for which strains are measured: i=1 for the x direction and i=2 for the y direction. The design variables to be determined in the optimization process are the material model parameters: Young’s modulus E and Poisson’s ratio υ. Design variables were constrained on both sides as shown as follows

(2) 800≤E≤1500

(3) 0.2≤υ≤0.5

The FEM simulation was made with the assumption that the model is static linear with isotropic material model. That limitation allows for determination of the model parameters for the first stage of the load and allows for the comparison of the results from the testing machine.

**Figure 10:**
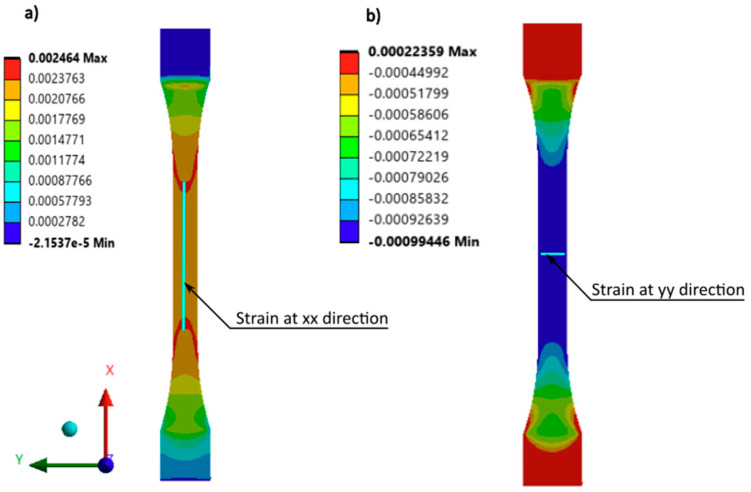
Engineering strain from FEM simulation for tensile load 285 N with the lines according to which mean value of strains are calculated according to deformations: (**a**) x-axis and (**b**) y-axis.

**Figure 11:**
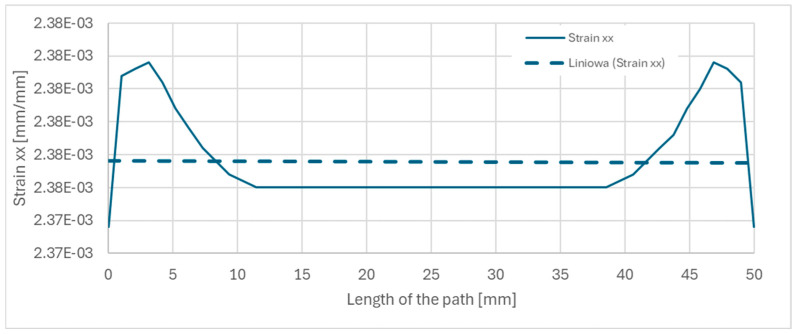
FEM strains change; the strains calculated on the 50 mm path—continuous line, the mean value of the strains—dashed line.

To ensure a consistent strain field across the analyzed area, the measurement length for the strains was determined using FEM simulations. The strains obtained from the simulation represent the average values calculated along the specified distances. The selected measurement distances were 50 mm for the x-direction (Figure 10a) and 8 mm for the y-direction (Figure 10b). Figure 11 illustrates the variation of strains in the x-direction with the depicted mean value considered in the optimization task.

**Figure 12:**
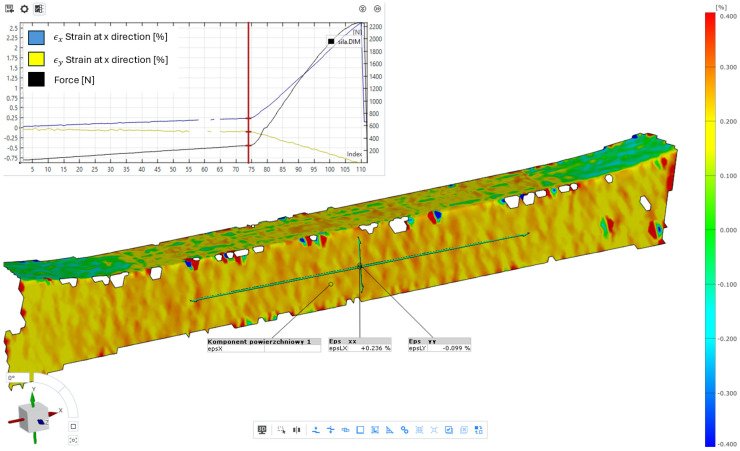
Strains in the x-direction for the stage marked by the red vertical line on the loading history plot, with the corresponding force of 285 N for the Tri-Hexagon infill type.

The first stage of the research was verification of the DIC measurements with the FEM simulation of the strains where in Figure 12 was depicted strains in the x direction at value 0.236%, which are strongly colinear with the deformation shown in Figure 10a) and plot Figure 11 with value 2.34 × 10^−3^ [mm/mm].

**Figure 13:**
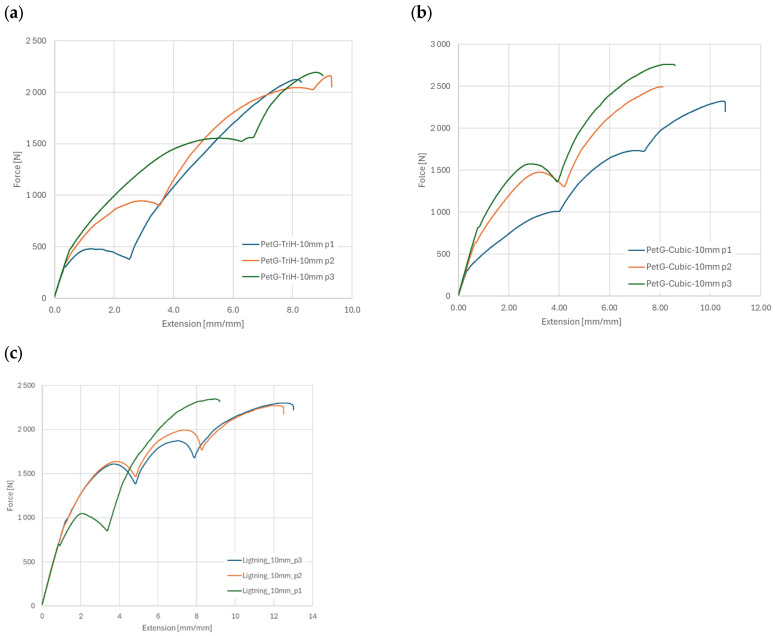
The change of the strains due to the tensile force registered on the testing machine for the 50% infill for the: (**a**) Tri-Hexagonal pattern, (**b**) Cubic pattern, (**c**) Lightning pattern.

The identification of the material parameters was made for three infill types: Tri-Hexagon, Lighting and Cubic (Figure 13). For each type of the infill, three probes were tested with the optimization methodology defined in Equation (1).

The extension change in function of the tensile force registered on the testing machine is depicted in Figure 13. It is worth noting that in tests of probes with a thickness of 4 mm compared to probes with a thickness of 10 mm, the graphs show abrupt changes in the tensile force, occurring in all samples regardless of the type of filling, which is due to the gradual degradation of the internal structure. Despite the changes in the registered force, its maximal value for the probes did not change a lot for the Tri-Hexagonal and Lightning pattern (Figure 13a,c) the change less than 5%, and the highest value is for the Cubic pattern with value 15% (Figure 13b). 

**Figure 14:**
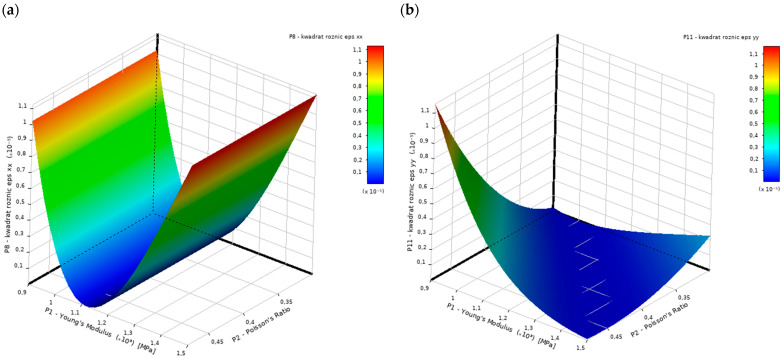
View of the response surface for the: (**a**) first objective function, (**b**) second objective function.

To optimize the problem, response surface optimization was employed, utilizing a surrogate model created through the Design of Experiments (DOE) methodology, specifically the Central Composite Design (CCD). The response surface depicted on Figure 14 was constructed using the Genetic Aggregation technique. The optimization process was carried out using the MOGA algorithm (Multi-Objective Genetic Algorithm), a variant of the widely recognized NSGA-II (Non-dominated Sorted Genetic Algorithm-II). The tools utilized for this task are part of the Design Exploration module provided in ANSYS software [50].

Table 6 shows the Young modulus determined during the tension test $E^{exp}$. The F value is the load for the final linear probe behaviour for which the $E^{exp}$ was determined and for which the strains $\varepsilon_{x}^{exp}$ and $\varepsilon_{y}^{exp}$ were taken by means of DIC method. Due to the optimization defined in Equation (1), the Young modulus $E^{inv}$ and Poisson ratio $\upsilon^{inv}$ are calculated.

The determined value of the Young’s modulus in Table 6 shows differences ranging from 7% for Cubic infill to less than 1% for Lightning infill. It should be noted that for the proposed method, only a one-time instance was used for determining the Young’s modulus. Additionally, what is most valuable is that instead of just one parameter of the material model, the Poisson ratio was determined as well.

The Young’s modulus values presented in Table 6 exhibit a pronounced correlation with the findings reported in [51]. Specifically, Young’s modulus for 20% infill is observed to be approximately 800 MPa, while for 100% infill, it reaches around 1800 MPa, as rigorously quantified in [52]. These results underscore the consistency of the mechanical properties across independent studies and highlight the reliability of the employed methodologies in assessing the elastic modulus of the material under varying infill conditions.

## 3. Discussion

This study investigated the mechanical properties of PET-G components fabricated via FDM 3D printing, focusing on the influence of key process parameters such as printing temperature, print speed, infill percentage, and infill pattern. The tensile strength tests, supported by digital image correlation (DIC) and finite element method updating (FEMU), provided insights into the mechanical behavior of the printed parts and highlighted the significant role of infill density and pattern selection in determining material performance. The results indicate that higher infill densities lead to greater tensile strength. As shown in Figure 6, the difference in breaking force between 10% and 100% infill is nearly 800 N. However, the increase in breaking force is marginal beyond 40% infill, suggesting an optimal trade-off between material usage and mechanical performance. Moreover, different infill patterns exhibited varying mechanical behaviors, with grid and cubic subdivision patterns providing superior strength compared to zigzag and gyroid patterns. Figure 8 illustrates that the optimal mechanical performance was achieved at mid-range printing temperatures (approximately 235 °C). While lower temperatures resulted in weaker layer bonding, excessively high temperatures led to material degradation and defects, negatively impacting the tensile strength of the printed specimens. Figure 7 shows that higher printing speeds adversely affected extrusion consistency, leading to reduced mechanical strength. The study suggests an optimal print speed of around 100 mm/s to balance print quality and production efficiency.

The research shows the problem with the lack of norms dedicated to 3D printed components. Utilizing the codes dedicated to plastics should be done carefully, with additional changes depending on the part for which additive technology is intended to be used. The study examined the influence of probe thickness, as depicted in Figure 9. It was observed that Young’s modulus decreases significantly with increasing thickness, stabilizing at around 8 mm. This finding underscores the importance of considering thickness as a crucial parameter in mechanical characterization.

The proposition of the connection of optical measurements with the finite element method analysis along with optimization shows the promising method of material model determination for the 3D printed components. The proposed method is based on determining the strains in the 3D-printed probe. It was shown that the method can be extended to the strains determined in various kinds of placement and length. The method allows for the determination of full model parameters but all necessary to determine the stiffness matrix for linear static simulation.

In most studies, the Poisson’s ratio is taken from material manufacturer specifications, typically reported within the range of 0.35 to 0.38. However, as evidenced by the research, the experimentally determined values exhibit a strong dependency on the infill pattern, indicating the necessity for precisely characterizing the mechanical response of 3D-printed components.

The research task can be formulated as a problem divided into more steps e.g., three sub-steps to capture the degradation of the material’s elastic parameters due to tensile load (Table 7). The first time instance corresponds to the end of the uniform deformation phase, the second is defined at the midpoint between the first and the peak destructive force, and the third corresponds to the maximum destructive force.

The data presented in Table 7 for increasing the load, even though showing the degradation of the material parameters, should not be treated as appropriate until a changed material model is introduced, which will be undertaken in the next step of the research.

**Figure 15:**
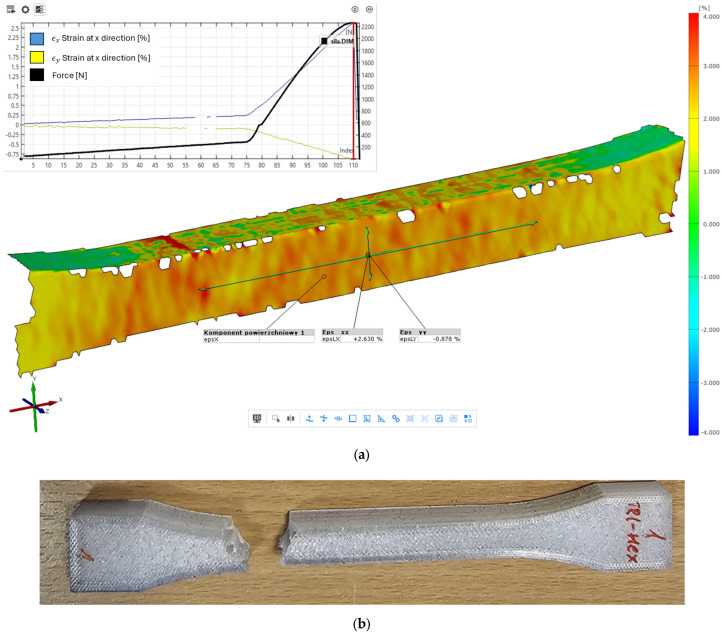
View strains in the x direction for Tri-Hexagon infill for the last step of tensile load right before breaking, marked by the red vertical line on the loading history plot, with the visible symptoms of the breaking place shown (**a**) and probes view after the test (**b**).

Most of the research dedicated to the determination of the mechanical properties of 3D-printed specimens suffers from the problem of inappropriate breaking placement (Figure 4). The location is close to the end of the radius R of the dog bone specimen (Figure 3). The phenomena can be precisely visible on the map of the x strains registered utilizing the DIC system (Figure 15) at the last step before the break where on the end of the radius rapid change of the strains, additionally unpredictable placement of the breaking point along the probe makes it difficult to use mechanical extensometers. The problem was noted in the literature [25] where increasing the radius was investigated. The problem is strictly connected with the failure mode which is related to the raster angle of the skin surfaces, the internal infill structure, and its density [43,53].

Incorporation of the optical measurements connected with the finite element model update and optimization enables precise determination of the material parameters. Contrary to the traditional material testing method, axial stretching, FEMU allows for determination during one test of more than one model parameter as was presented in the research where the Young modulus and Poisson ratio were properly determined.

Internal porosity and interlayer bonding are critical to mechanical properties. While the presented study does not consider these factors, future research will use advanced non-destructive techniques, such as X-ray CT, to correlate porosity and bonding quality with material strength. 

### Practical Applications

This study employs an advanced integration of Digital Image Correlation (DIC) and Finite Element Method Updating (FEMU) to rigorously quantify the Young’s modulus and Poisson’s ratio of PET-G specimens fabricated via FDM 3D printing. The results elucidate the complex interdependencies among key process parameters—including extrusion temperature, infill density, raster orientation, and layer height—and their respective influences on the resultant mechanical properties. By leveraging full-field strain mapping through DIC and optimizing constitutive parameters via inverse FEM-based identification, this approach offers a more comprehensive material characterization than traditional tensile testing methods, which inherently assume homogeneity in the cross-section. The implications of these findings are significant for industrial applications where precision in material performance prediction is paramount. By systematically optimizing process parameters, engineers can enhance the reliability of printed components while simultaneously minimizing prototyping iterations and material waste. The detailed insights into the mechanical properties of various infill architectures enable the development of application-specific strategies to optimize structural efficiency, reducing mass without compromising mechanical integrity. Furthermore, the proposed methodological framework extends seamlessly to the study of nonlinear and time-dependent behaviours, thereby addressing the stringent safety and durability requirements of aerospace, biomedical, and high-performance engineering applications. In addition to its immediate industrial relevance, this methodology contributes to the broader discourse on standardization within additive manufacturing. Conventional polymer testing standards fail to accommodate the intrinsic heterogeneities of FDM-printed components, particularly in terms of interlayer adhesion and localized strain distribution. By integrating DIC with FEMU-based material characterization, researchers and practitioners can establish more accurate predictive models, refine failure criteria, and facilitate the adoption of performance-driven material selection strategies. Ultimately, this refined approach fosters advancements in the engineering and certification of 3D-printed components, ensuring their reliability and cost-effectiveness across diverse technological domains.

## 4. Conclusions

This study demonstrated the critical influence of printing parameters on the mechanical performance of PET-G components fabricated through FDM. Key findings include:

- Infill density significantly impacts tensile strength, with optimal mechanical properties observed at densities above 40%;
- Infill pattern selection affects mechanical performance, with grid and cubic subdivision patterns offering higher strength than other designs;
- Printing temperature plays a crucial role, with mid-range temperatures (~235 °C) providing the best layer adhesion and mechanical properties;
- Printing speed should be optimized at around 100 mm/s to prevent material inconsistencies;
- Probe thickness influences Young’s modulus, highlighting the need for standardized thickness selection in mechanical testing;
- Inverse problem approaches (FEMU & DIC) offer a precise methodology for material property determination, enabling accurate characterization of 3D-printed components.

The findings underscore the urgent need for dedicated standards in the mechanical characterization of 3D-printed materials. While traditional plastic testing standards provide a foundation, they do not fully account for the anisotropic and layer-dependent nature of FDM-produced components. Integrating optical measurements with FEM-based methodologies enables a more precise depiction of material performance, facilitating better predictive models for structural applications. Standardized testing protocols will ensure the reliability of 3D-printed parts, reducing variability and enhancing quality control across the industry. Furthermore, this research contributes to additive manufacturing by demonstrating how systematic parameter control improves mechanical performance. By refining computational simulations and leveraging advanced measurement techniques, the reliability and efficiency of 3D-printed components can be significantly enhanced. These advancements will accelerate the adoption of FDM printing in high-precision engineering, ensuring consistency, durability, and performance across various industries.

## Figures and Tables

**Table 1:** PET-G printing material characteristics [48].

Description	Unit	Value
Print temperature	[°C]	220–250
Printer bed temperature	[°C]	60–70
Heat Deflection Temperature	[°C]	70
Tensile Strength	[MPa]	50
Flexural Strength	[MPa]	69
Impact Strength	[kJ/m^2^]	8.1
Colour	X	Transparent
Filament diameter	[mm]	1.75 ± 0.05
Filament roundness	[mm]	±0.02
Density	$\left[\frac{g}{{cm}^{3}}\right]$	1.29

**Table 2:** Extrusion test with constant extrusion velocity.

No.	Temperature	S1	S2	S3	S4	S5	S6	S7	S8	S9	S10	Mean Value
	°C	g										
1	210	0.3	0.27	0.28	0.28	0.29	0.29	0.28	0.28	0.28	0.28	0.283
2	215	0.28	0.29	0.29	0.28	0.28	0.28	0.27	0.28	0.28	0.29	0.282
3	220	0.29	0.3	0.3	0.28	0.29	0.29	0.27	0.29	0.29	0.28	0.288
4	225	0.29	0.29	0.3	0.3	0.28	0.28	0.29	0.3	0.3	0.27	0.29
5	230	0.28	0.29	0.3	0.28	0.29	0.3	0.29	0.28	0.3	0.29	0.29
6	240	0.29	0.3	0.29	0.3	0.3	0.29	0.29	0.3	0.28	0.3	0.294
7	245	0.3	0.3	0.3	0.29	0.31	0.3	0.29	0.31	0.28	0.29	0.297
8	250	0.31	0.3	0.31	0.31	0.3	0.29	0.31	0.31	0.3	0.3	0.304

**Table 3:** Extrusion test with constant extrusion temperature.

No.	Extrusion Speed	S1	S2	S3	S4	S5	S6	S7	S8	S9	S10	Mean Value
X	mm/min	g										
1	150	0.29	0.29	0.3	0.3	0.28	0.28	0.29	0.3	0.3	0.27	0.29
2	200	0.28	0.27	0.28	0.28	0.28	0.27	0.28	0.26	0.28	0.28	0.276
3	250	0.28	0.28	0.27	0.28	0.28	0.27	0.28	0.27	0.29	0.28	0.278
4	300	0.27	0.26	0.27	0.28	0.28	0.27	0.27	0.27	0.27	0.27	0.271
5	350	0.26	0.25	0.27	0.26	0.27	0.26	0.27	0.27	0.28	0.27	0.266
6	400	0.26	0.25	0.26	0.26	0.26	0.25	0.25	0.26	0.27	0.24	0.256
7	450	0.24	0.24	0.25	0.25	0.24	0.23	0.25	0.24	0.25	0.25	0.244
8	500	0.22	0.21	0.22	0.23	0.22	0.23	0.22	0.21	0.22	0.23	0.221

**Table 4:** Testing samples characteristics.

Number	Layer Height	Infill Type	Fill Percentage	Print Temperature	Print Speed	Number of Samples
	[mm]	X	[%}	[°C]	[mm/s]	[pcs]
1	0.2	Concentric	50	235	100	6
2	0.2	Cross 3d	50	235	100	6
3	0.2	Cross	50	235	100	6
4	0.2	Cubic	50	235	100	6
5	0.2	Cubic Subdivision	50	235	100	6
6	0.2	Grid	50	235	100	6
7	0.2	Gyroid	50	235	100	6
8	0.2	Lightning	50	235	100	6
9	0.2	Lines	50	235	100	6
10	0.2	Octet	50	235	100	6
11	0.2	Quatercubic	50	235	100	6
12	0.2	Triangles	50	235	100	6
13	0.2	Tri-Hexagon	50	235	100	6
14	0.2	ZigZag	50	235	100	6
15	0.2	Grid	10	235	100	6
16	0.2	Grid	20	235	100	6
17	0.2	Grid	30	235	100	6
18	0.2	Grid	40	235	100	6
19	0.2	Grid	60	235	100	6
20	0.2	Grid	70	235	100	6
21	0.2	Grid	80	235	100	6
22	0.2	Grid	90	235	100	6
23	0.2	Grid	100	235	100	6
24	0.12	Grid	50	235	100	6
25	0.16	Grid	50	235	100	6
26	0.28	Grid	50	235	100	6
27	0.32	Grid	50	235	100	6
28	0.2	Grid	50	220	100	6
29	0.2	Grid	50	225	100	6
30	0.2	Grid	50	230	100	6
31	0.2	Grid	50	240	100	6
32	0.2	Grid	50	245	100	6
33	0.2	Grid	50	250	100	6
34	0.2	Grid	50	235	50	6
35	0.2	Grid	50	235	150	6

**Table 5:** Main printing parameters chosen in the survey.

Description	Unit	Value
Thickness of the printed layer	[mm]	0.2
Infill type	X	Table 3
Filling density	[%]	Table 3
Print temperature	[°C]	Table 3
Printer bed temperature	[°C]	60
Print speed	[mm/s]	Table 3
Number of samples per test	[pcs]	6

**Table 6:** Invers problem input data and results.

Infill Type	Probe No.	$E^{exp}$	F	$\varepsilon_{x}^{exp}$	$\varepsilon_{y}^{exp}$	$E^{inv}$	$\upsilon^{inv}$
		MPa	N	%	%	MPa	--
Cubic	1	1130	285	0.282	−0.108	1014	0.382
	2	1130	278	0.259	−0.101	1076.5	0.389
	3	1140	278	0.26	−0.088	1071.6	0.3386
	average	1133	--	--	--	1054	0.37
Lightning	1	984	255	0.262	−0.111	977.58	0.4238
	2	948	253	0.254	−0.14	993.08	0.4797
	3	959	255	0.3	−0.132	901.1	0.4661
	average	964	--	--	--	957	0.456
Tri-Hex	1	1140	284	0.239	−0.087	1189	0.3645
	2	1120	290	0.246	−0.036	991.13	0.3
	3	1090	285	0.295	−0.088	970	0.3
	average	1117	--	--	--	1050	0.32

**Table 7:** Inverse problem input data and results for the loads sub-steps.

Infill Type	Load Sub-Step	$E^{exp}$	F	$\varepsilon_{x}^{exp}$	$\varepsilon_{y}^{exp}$	$E^{inv}$	$\upsilon^{inv}$
		MPa	N	%	%	MPa	--
Cubic	1	1130	285	0.282	−0.108	1014	0.382
	2	1130	1805	1.812	−0.542	1002.3	0.30015
	3	1140	2343	3.193	−1.081	900.72	0.3
Lightning	1	984	255	0.262	−0.111	977.58	0.47
	2	948	1625	1.801	−0.65	902	0.47
	3	959	2257	3.663	−0.961	901	0.38
Tri-Hex	1	1140	284	0.239	−0.087	1189	0.3645
	2	1120	1619	1.695	−0.474	991.02	0.3
	3	1090	2253	2.899	−0.631	900	0.25

## Data Availability

The original contributions presented in the study are included in the article, further inquiries can be directed to the corresponding author.
